# Laparoscopic Splenectomy for a Completely Infarcted Wandering Spleen After Prolonged Positional Abdominal Pain: A Case Report and Focused Literature Review

**DOI:** 10.1002/ccr3.73607

**Published:** 2026-09-24

**Authors:** Toufic Awwad, Ahmad Jradi, Riwa Deghaim, Mohamad Khalife

**Affiliations:** ^1^ Department of Surgery American University of Beirut Medical Center Beirut Lebanon

**Keywords:** laparoscopic splenectomy, splenic infarction, splenic torsion, thrombocytopenia, wandering spleen

## Abstract

A 19‐year‐old woman had a 7–8‐year history of positional abdominal pain, a mobile mass and transient thrombocytopenia. Computed tomography showed a completely infarcted wandering spleen with pedicle torsion. Fully laparoscopic splenectomy succeeded despite total infarction. Recognizing this triad may prompt earlier imaging and does not preclude minimally invasive management.

## Key Clinical Message

Wandering spleen should be considered in young patients with recurrent positional abdominal pain, a mobile abdominal mass, and unexplained fluctuating thrombocytopenia. Earlier recognition may permit splenopexy before infarction; once infarction is established, laparoscopic splenectomy remains feasible in selected haemodynamically stable patients.

## 1 Introduction

Wandering spleen is displacement of the spleen from its normal anatomical position because of absent or lax suspensory ligaments. The spleen is consequently supported mainly by an elongated vascular pedicle, predisposing it to torsion, venous congestion, ischaemia and infarction [1]. Congenital cases result from abnormal dorsal mesogastric development, whereas acquired ligamentous laxity has been associated with multiparity and splenomegaly. Among adults, it predominantly affects women of reproductive age [1]. Presentation ranges from an incidental mobile mass to acute infarction. Intermittent torsion and spontaneous detorsion may produce recurrent positional symptoms, making recognition difficult before an acute complication develops [1, 2, 3]. Most published reports emphasize either acute abdominal presentation, nonspecific recurrent pain or infarction managed by open surgery. We report a young nulliparous woman with prolonged movement‐triggered and posture‐relieved pain, a mobile lower abdominal mass, transient thrombocytopenia and complete splenic infarction successfully managed laparoscopically. A focused literature review is included to clarify the diagnostic clues and operative factors that supported a minimally invasive approach.

## 2 Case History/Examination

A 19‐year‐old nulliparous woman presented with recurrent sharp abdominal pain that had begun 7–8 years earlier. The episodes were reproducibly triggered by rising from bed, jogging or turning to one side and improved after postural adjustment. She denied nausea, vomiting, fever or constipation. She had also noticed a freely mobile lower abdominal mass. Details of previous formal assessment for these episodes were unavailable. She reported easy bruising during contact sports and heavy menstrual bleeding. Routine testing 3–4 months earlier had shown a platelet count of 60 × 10^9^/L; no contemporaneous imaging or documented hematological evaluation was available. Her medical and surgical history was otherwise unremarkable.

She was haemodynamically stable. The abdomen was soft and non‐tender, with a smooth, freely mobile mass that was dull to percussion in the left lower quadrant. Scattered bruises were present. White blood cell count was 7.3 × 10^9^/L, hemoglobin 14 g/dL, haematocrit 43%, platelet count 154 × 10^9^/L and creatinine 0.6 mg/dL.

## 3 Methods (Differential Diagnosis, Investigations and Treatment)

Contrast‐enhanced computed tomography showed absence of the spleen from the left upper quadrant and an ectopic spleen in the left lower quadrant. Complete absence of parenchymal enhancement was consistent with total infarction, and a whirl sign demonstrated torsion of the vascular pedicle. Given the radiological evidence of pedicle torsion and complete splenic infarction, urgent laparoscopic exploration was undertaken.

**FIGURE 1:**
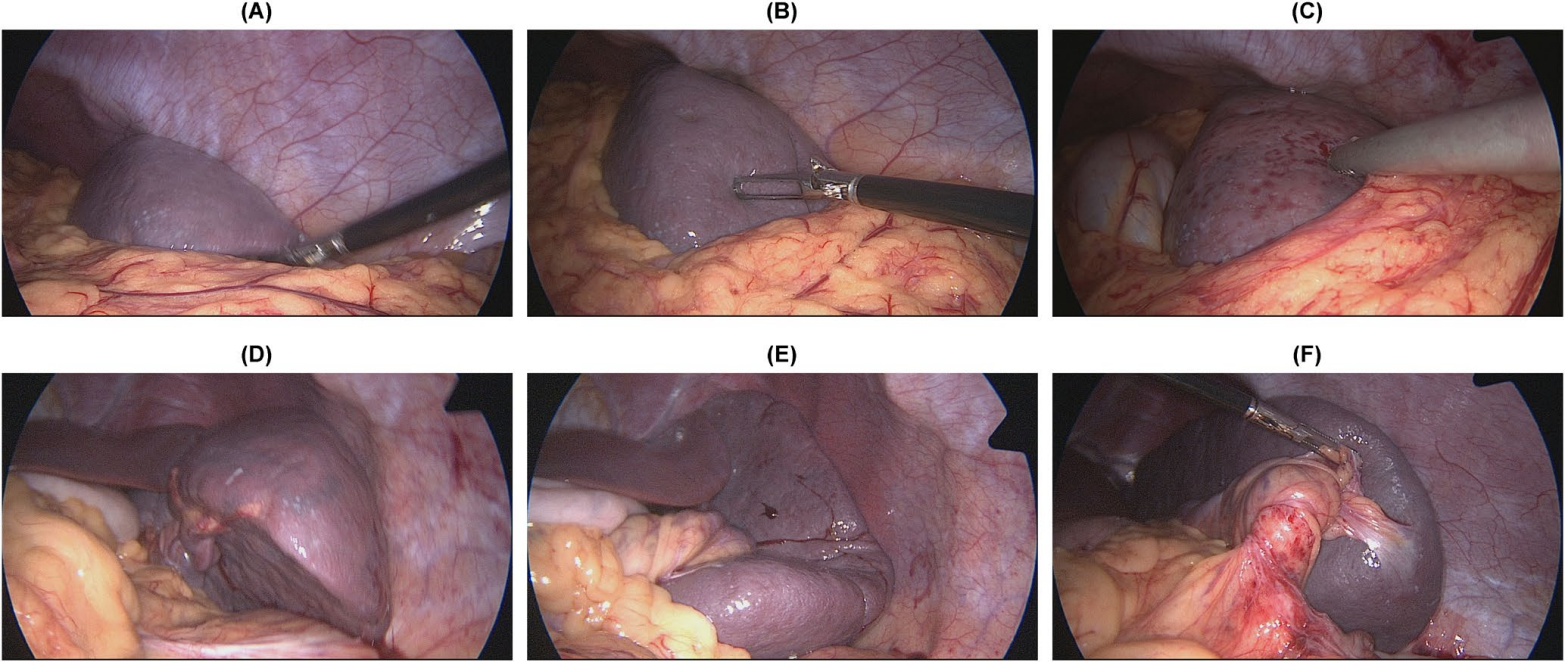
Intraoperative laparoscopic findings. (A) Freely mobile ectopic spleen in the lower abdominal cavity. (B) Laparoscopic manipulation demonstrating the mobility of the ectopic spleen. (C) Congested infarcted splenic surface with petechial and haemorrhagic changes. (D) Tightly twisted, rope‐like vascular pedicle adjacent to the congested spleen. (E) Elongated vascular pedicle following detorsion. (F) Exposure and laparoscopic control of the splenic pedicle before division.

The patient was placed supine under general anesthesia, and pneumoperitoneum was established. Laparoscopic ports were positioned to triangulate the ectopic spleen in the lower abdomen. At laparoscopy, the spleen was freely mobile and showed extensive ischaemic/infarcted change, with a twisted elongated vascular pedicle (Figure 1). Because the spleen was non‐viable, laparoscopic splenectomy was performed. The twisted pedicle was carefully detorsed to restore its anatomical orientation and facilitate safe identification of the hilar structures; no macroscopic reperfusion occurred. After confirming that the pancreatic tail was clearly excluded from the intended staple line, the elongated splenic pedicle was controlled and divided en bloc close to the splenic hilum using an endoscopic linear stapler with a vascular load. The staple line was inspected under reduced pneumoperitoneum, and complete hemostasis was confirmed. The infarcted spleen was placed in an endoscopic retrieval bag and extracted intact, without morcellation, through a small extension of the umbilical port site (Figure 1). No drain was placed.

## 4 Conclusions and Results (Outcome and Follow‐Up)

The spleen weighed 224 g and measured 15 × 14 × 3 cm. Histopathology showed diffuse vascular congestion and infarction without malignancy. Recovery was uncomplicated, and the patient was discharged on postoperative day 3. Post‐splenectomy vaccination and oral antibiotic prophylaxis were initiated according to the institutional asplenia protocol. The patient was counseled regarding the lifelong risk of overwhelming post‐splenectomy infection and the need for urgent medical assessment in the event of fever [4, 5]. At the 2‐week postoperative follow‐up, she remained clinically well and reported complete resolution of her previous positional abdominal pain.

The favorable postoperative course supports laparoscopic splenectomy as a feasible option in appropriately selected haemodynamically stable patients with an infarcted wandering spleen.

## 5 Discussion

The patient's young age and nulliparity favor a congenital etiology. Her prolonged movement‐related symptoms are compatible with intermittent torsion and detorsion of the elongated vascular pedicle. Because the episodes were self‐resolving and relieved by postural adjustment, wandering spleen may remain clinically unrecognized until irreversible vascular compromise develops, particularly when no prior imaging is available.

The earlier platelet count of 60 × 10^9^/L may reflect temporary congestion and splenic sequestration during episodic torsion. However, this mechanism cannot be established from a single historical value because no imaging, peripheral blood smear, or hematological evaluation was documented during that episode. Heavy menstrual bleeding and bruising may also have had other explanations. The thrombocytopenia should therefore be interpreted as a possible diagnostic clue rather than proof of intermittent hypersplenism. A proposed mechanism is that partial pedicle torsion transiently kinks the splenic vein, producing passive venous congestion and temporary intrasplenic platelet pooling that resolves once the pedicle untwists; this remains hypothetical in the absence of contemporaneous imaging or hematological evaluation. A similar association between recurrent pain, wandering spleen, and thrombocytopenia has been described [6].

Computed tomography is central to diagnosis because it demonstrates the ectopic spleen, vascular‐pedicle torsion and parenchymal perfusion [7]. When the spleen remains viable, splenopexy is preferred to preserve immune function [8]. Established infarction requires splenectomy. Open splenectomy has commonly been used in acutely infarcted or markedly congested wandering spleens [9, 10], whereas laparoscopic splenectomy has also been successfully performed in selected patients [11]. In this case, haemodynamic stability, manageable splenic dimensions, adequate pedicle exposure, secure laparoscopic vascular control and intact specimen retrieval supported the minimally invasive approach. A minimally invasive approach to an infarcted wandering spleen is most appropriate when several conditions are met: haemodynamic stability without septic shock or active hemorrhage; non‐massive splenic enlargement that permits manipulation and retrieval; absence of dense inflammatory adhesions; and adequate exposure to isolate and control the vascular pedicle safely. Complete infarction should therefore not be regarded as an automatic indication for laparotomy; the approach should be individualized to splenic size, congestion, pedicle anatomy and the ability to obtain safe control.

## 6 Focused Literature Review and Clinical Significance

This focused narrative review was reported with reference to applicable PRISMA 2020 principles [12] to improve transparency in record identification, deduplication, screening and selection; it was not designed as a formal systematic review or meta‐analysis. PubMed/MEDLINE (title/abstract fields) and Scopus (title, abstract and keywords) were searched from database inception through 19 August 2026. The PubMed strategy was: “wandering spleen”[Title/Abstract] AND (((laparoscop*[Title/Abstract]) AND (torsion[Title/Abstract] OR infarct*[Title/Abstract])) OR thrombocytopen*[Title/Abstract] OR (((recurrent[Title/Abstract] OR intermittent[Title/Abstract] OR positional[Title/Abstract]) AND “abdominal pain”[Title/Abstract]))) AND english[lang]. The Scopus strategy was: TITLE‐ABS‐KEY(“wandering spleen” AND ((laparoscop* AND (torsion OR infarct*)) OR thrombocytopen* OR ((recurrent OR intermittent OR positional) AND “abdominal pain”))). English‐language limits were applied; no publication‐date restriction was used. Eligible records were case reports, case series or reviews providing direct evidence on recurrent, intermittent or positional symptoms; thrombocytopenia or hypersplenism; torsion, ischaemia, infarction or splenic viability; operative management; or focused multi‐patient evidence. Records primarily addressing unrelated anatomy or clinical contexts, adjacent pathology, viable‐spleen techniques outside the focused comparison, or providing insufficient wandering‐spleen‐specific information were excluded. Records were combined and deduplicated hierarchically by PMID, DOI and normalized title. One author (RD) screened titles and abstracts against the prespecified criteria. Overall, 275 records were identified (113 from PubMed/MEDLINE and 162 from Scopus). After removal of 90 duplicates, 185 unique records were screened; 117 were excluded and 68 were retained for focused qualitative synthesis. Retained records were grouped into recurrent, intermittent or positional pain; thrombocytopenia or hypersplenism; torsion, infarction and operative management; and review or multi‐patient evidence. Because the evidence consisted predominantly of heterogeneous case reports, no formal risk‐of‐bias tool or quantitative synthesis was applied; clinical relevance, comparability and completeness of reporting were considered during narrative interpretation.

Recent reports (2024–2025) have continued to describe wandering spleen complicated by pedicle torsion and infarction requiring splenectomy [13, 14], while thrombocytopenia attributed to splenic congestion or sequestration has likewise been reported with wandering spleen and torsion [15, 16, 17], consistent with the pattern observed in the present case. Previous reports have separately documented recurrent abdominal pain with thrombocytopenia [6], delayed recognition over approximately a decade culminating in torsion, infarction and open splenectomy [18], and minimally invasive splenectomy for an infarcted wandering spleen in an older patient or during pregnancy [11, 19]. By contrast, the present case combines a reproducible movement‐triggered and posture‐relieved pain pattern beginning in early adolescence, a freely mobile mass, transient marked thrombocytopenia, radiologically complete infarction and fully laparoscopic splenectomy. Its educational contribution is therefore the recognizable diagnostic pattern and the explicit selection rationale for laparoscopy, rather than rarity alone.

Taken together, these features suggest a practical diagnostic triad: recurrent positional abdominal pain, a mobile abdominal mass and unexplained fluctuating thrombocytopenia. Recognition of this pattern may justify earlier cross‐sectional imaging and potentially permit splenic preservation before irreversible infarction develops.

## 7 Limitations

This report has limitations inherent to a single case. Postoperative follow‐up was short, and the degree of pedicle rotation was not quantified. Non‐viability was supported by absent enhancement on computed tomography and histological confirmation; intraoperatively, no macroscopic reperfusion was observed following detorsion. The focused literature review was narrative rather than systematic and was intended to contextualize clinical decision‐making rather than estimate comparative outcomes.

Wandering spleen should be considered in young patients with recurrent positional abdominal pain and a mobile abdominal mass, particularly when accompanied by unexplained fluctuating thrombocytopenia. Earlier recognition may permit splenopexy before infarction. When infarction is established, laparoscopic splenectomy may remain feasible in haemodynamically stable patients with manageable splenic size, favorable exposure and safe pedicle control. Complete infarction should not automatically mandate laparotomy. Post‐splenectomy vaccination, antimicrobial guidance and education regarding overwhelming post‐splenectomy infection remain essential.

## Author Contributions

**Toufic Awwad:** investigation, writing – original draft, writing – review and editing, visualization, validation, methodology, conceptualization, resources. **Ahmad Jradi:** investigation, writing – original draft, writing – review and editing, visualization, validation, methodology, resources. **Riwa Deghaim:** investigation, writing – review and editing, visualization, validation, methodology, resources. **Mohamad Khalife:** conceptualization, investigation, writing – review and editing, validation, visualization, methodology, supervision.

## Funding

The authors have nothing to report.

## Ethics Statement

This report describes a single retrospective case. In accordance with the institutional policies of the American University of Beirut Medical Center, formal Institutional Review Board approval is not required for a single‐patient case report, as it does not constitute human subjects research. All identifying information was removed to protect patient confidentiality. This report was prepared in accordance with the CARE guidelines [20].

## Consent

Written informed consent was obtained from the patient for publication of the de‐identified clinical information and accompanying images. A copy of the written consent is available for review by the Editor‐in‐Chief of this journal on request.

## Conflicts of Interest

The authors declare no conflicts of interest.

## Data Availability

The data that support the findings of this study are available from the corresponding author upon reasonable request.
